# Improving image contrast and material discrimination with nonlinear response in bimodal atomic force microscopy

**DOI:** 10.1038/ncomms7270

**Published:** 2015-02-10

**Authors:** Daniel Forchheimer, Robert Forchheimer, David B. Haviland

**Affiliations:** 1 Department of Applied Physics, Section for Nanostructure Physics, Royal Institute of Technology (KTH), SE-106 91 Stockholm, Sweden; 2 Division of Information Coding, Department of Electrical Engineering, Linköping University, SE- 581 83 Linköping, Sweden

## Abstract

Atomic force microscopy has recently been extented to bimodal operation, where increased image contrast is achieved through excitation and measurement of two cantilever eigenmodes. This enhanced material contrast is advantageous in analysis of complex heterogeneous materials with phase separation on the micro or nanometre scale. Here we show that much greater image contrast results from analysis of nonlinear response to the bimodal drive, at harmonics and mixing frequencies. The amplitude and phase of up to 17 frequencies are simultaneously measured in a single scan. Using a machine-learning algorithm we demonstrate almost threefold improvement in the ability to separate material components of a polymer blend when including this nonlinear response. Beyond the statistical analysis performed here, analysis of nonlinear response could be used to obtain quantitative material properties at high speeds and with enhanced resolution.

## Abstract (web summary)

Bimodal atomic force microscopy is a promising approach in obtaining high-quality image contrast and material property mapping. Here, the authors show that by considering nonlinear response in bimodal atomic force microscopy, significant improvements in image contrast and material discrimination can be achieved.

## Graphical abstract

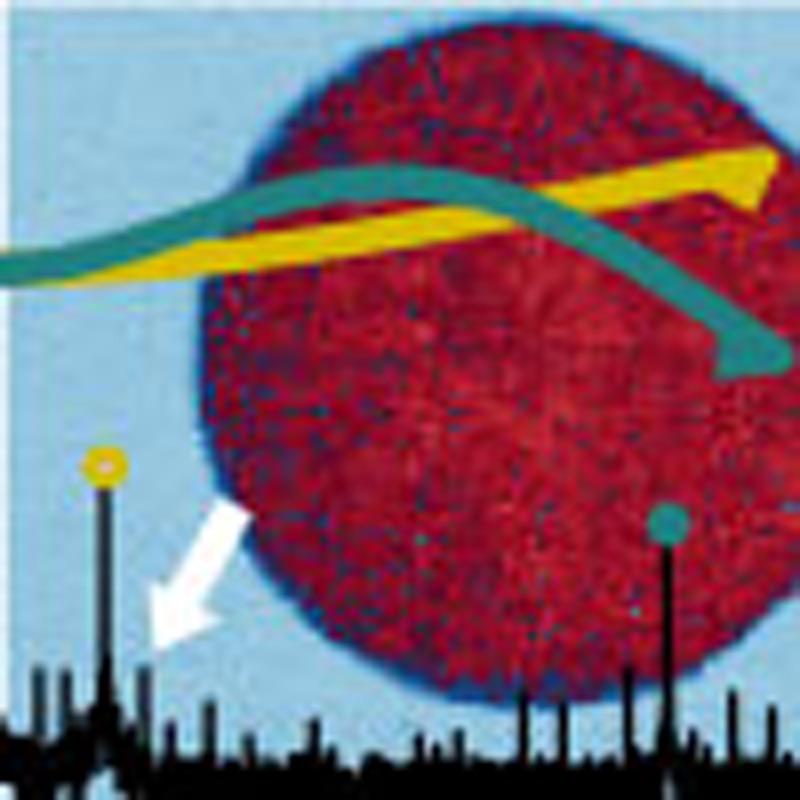

Detailed analysis of cantilever dynamics in atomic force microscopy (AFM) allows one to go beyond simple topography mapping to study the physical properties of a material's surface^1^,^2^. The recent development of multifrequency AFM facilitates such analysis by measuring response at harmonics^3^,^4^, in a continuous frequency band^5^, at discrete tones near one resonance^6^ and at two or more flexural resonances^7^,^8^,^9^. This later so-called bimodal AFM has demonstrated increased material contrast^10^ and material property mapping^11^, but the limited number of measured signals restricts mapping to parameters of very simple tip–surface interaction models. Additional signals are available if one measures response at the intermodulation products or mixing frequencies of the bimodal drive^12^,^13^,^14^ but the utility of these extra signals for imaging has never been demonstrated. We show that image contrast at mixing frequencies can be larger than at drive frequencies, and we use Fisher's linear discriminant analysis^15^ on the collective multifrequency response to demonstrate quantitative improvement of material contrast.

Traditional modes of dynamic AFM derive each image point from two measured quantities: the amplitude and phase of cantilever oscillation at the frequency of the drive. Feedback adjusts the probe height so as to keep the oscillation amplitude constant and this constant amplitude height image is typically interpreted as surface topography. Changes in the phase indicate a variation of material property. Assuming sinusoidal motion, the phase image can be interpreted as a map of energy dissipation^16^ and for many years this phase image was the only information the AFM operator had to understand the physical character of the surface.

To improve the AFM's ability to sense material properties one must increase the number of measured signals. Multifrequency AFM^17^ approaches this task in the frequency domain by exciting and measuring the response of the cantilever at many frequencies in the time required to record one pixel, where each frequency provides two observable quantities. Bimodal AFM^7^, where the cantilever is excited at the resonant frequencies of two different eigenmodes, has been extended to multiple eigenmodes^9^,^18^,^19^. This approach gives more signals, but it requires broadband detection. With the photodetectors used in today’s AFMs it is typically not feasible to measure more than the first few modes.

Rather than extending the number of eigenmodes, more information can be obtained in the limited detection bandwidth by capturing response at non-driven frequencies. When the cantilever is driven with two tones at frequencies *f*_1_ and *f*_2_, the nonlinear tip–surface interaction generates harmonics and so-called intermodulation or mixing products at frequencies


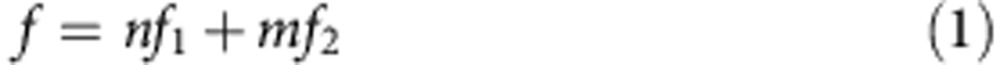


where *n* and *m* are integers (positive or negative), and |*n*|+|*m*| is the order of the intermodulation product^6^. Below we show that this nonlinear response in bimodal AFM can be utilized to produce higher image contrast than the linear response.

## Results

### Free and engaged cantilever dynamics

An AFM cantilever was inertially excited using a shaker piezo driven with a superposition of two pure tones at *f*_1_=78.5 and *f*_2_=500.5 kHz, very close to the first two flexural eigenfrequencies. The excitation was not exactly on resonance, as the second resonance frequency is not a rational fraction of the first. True bimodal oscillation is therefore incommensurate, with each oscillation at the first flexural eigenfrequency slightly different from the previous. To ensure periodicity in the measurement window or pixel time *T*, the two drive frequencies were chosen to be integer multiples of one base frequency, Δ*f*=1/*T*=0.5 kHz. This choice avoids spectral leakage and enables the measurement of both amplitude and phase at intermodulation frequencies. When free from the surface, the drive signal was adjusted such that the amplitude of tip motion at the first eigenmode was 17 nm (290 mV). The free amplitude at the second eigenmode was adjusted to be 20% of the voltage amplitude at the first mode, as measured by the photodetector. Using the theoretical difference in detector responsivity, we estimate the free oscillation amplitude of tip motion at the second mode to be about 1 nm (see Methods for calibration details).

**Figure 1:**
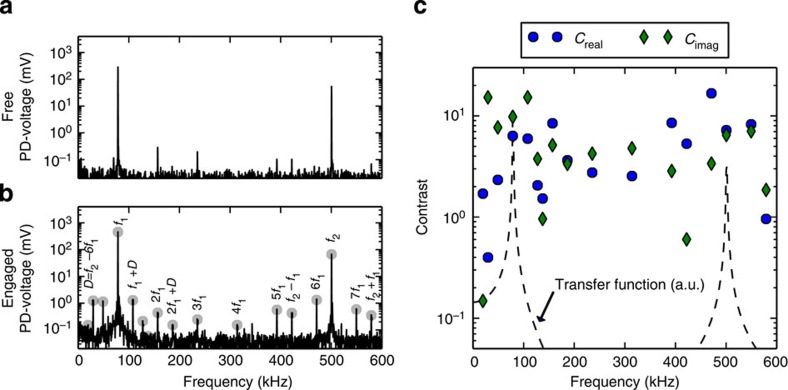
Photodiode (PD) voltage amplitude spectra and contrast metric. Spectrum **a** is the free response amplitude and **b** the engaged response amplitude. The engaged spectrum contains many additional mixing frequencies due to the nonlinear tip–surface interaction. Grey circles mark frequencies used for imaging and analysis. Spectra were obtained in a time window of *T*=2 ms. (**c**) The contrast metric given in Table 1 plotted together with the approximate response function of each eigenmode. Although the force responsivity and PD amplitude are much higher on resonance, off resonance contrast is excellent, in many cases better than on resonance.

The spectrum of the freely oscillating cantilever showed weak mixing products and harmonics (see Fig. 1a), which we attribute to nonlinearity in either the drive electronics, the excitation piezo, the photodetector or the digital sampling electronics. This ‘background’ nonlinearity can limit the ability to precisely reconstruct tip–surface force, but it will not effect the statistical analysis used here, which does not require knowledge of the physical theory connecting tip–surface force with cantilever motion. When the oscillating cantilever engaged a surface, many new mixing products appeared above the background (compare Fig. 1a,b) clearly due to the tip–surface interaction. The response at 6*f*_1_ is stronger than other harmonics as this frequency falls closest to the second eigenfrequency of the cantilever. The difference frequency


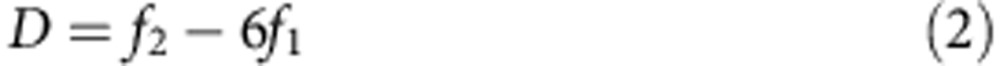


is a factor in many of the stronger intermodulation products (see Table 1).

### Imaging

**Figure 2:**
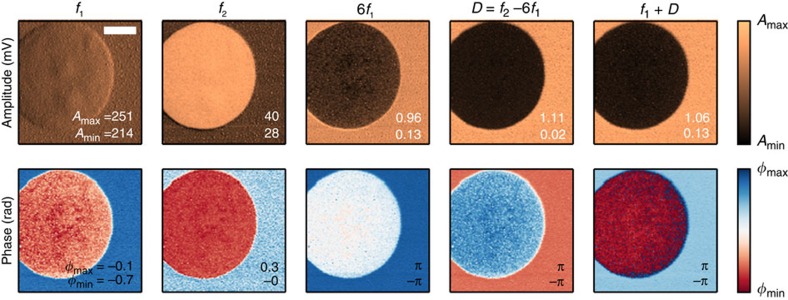
Subset of amplitude and phase images of PS –LDPE. White scale bar is 500 nm, total scan size was 2 μm × 2 μm. Numbers in bottom right corners show the limits of the colour scales *A*_max_, *A*_min_ and *ø*_max_, *ø*_min_ for each amplitude and phase image respectively. See Supplementary Table I for amplitude ratios between PS and LDPE.

We measured the response at the dominant peaks in the spectra, listed in Table 1, while scanning the surface of a blend of polystyrene and low-density polyethylene (PS–LDPE), Fig. 2. A multifrequency lockin amplifier^20^ captured the amplitude and phase at all frequencies in real time during a single scan. As LDPE is much softer than PS (~0.1 GPa versus ~2 GPa^21^), excellent contrast is seen in the amplitude and phase images at all frequencies, except the amplitude at *f*_1_ used for feedback (see Supplementary Table I).

To quantify image contrast at different frequencies we converted the amplitude and phase into their real and imaginary quadratures (Supplementary Fig. 1) and made image histograms of these quantities (Supplementary Fig. 2). Representation of the response data in a Cartesian plane avoids ambiguity associated with the wrapping of phase, and it allowed all histograms to have the same physical units of detector voltage. The image histograms contained one peak corresponding to PS and one peak for LDPE. We fit the histograms to a binormal distribution (see Methods) and define a contrast metric,


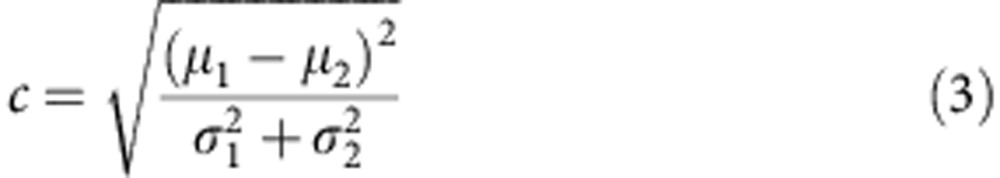


Where, *μ* and *σ*^2^ are the mean and variance of the two normal distributions. Two contrast values *c*_real_ and *c*_imag_ were calculated at each frequency, from the real and imaginary quadrature images, respectively. Although the response amplitudes at the driven frequencies *f*_1_ and *f*_2_ were orders of magnitude larger than at harmonics and mixing tones, the latter often showed higher contrast (see Fig. 1c and Table 1). In other words, discrimination of the two polymer components was greater at weakly responding mixing frequencies than at strongly responding drive frequencies. A second sample consisting of a blend of polystyrene and polymethylmethacrylate (PS–PMMA) showed lower contrast than PS–LDPE at all frequencies (see Supplementary Figs 3,4 and Supplementary Tables II,III), which was expected as the two polymers have similar elastic moduli^22^. For this sample the largest contrast was found at *f*_1_, with several mixing frequencies having higher contrast than that at *f*_2_.

### Linear discriminant analysis

One scan generates a 34-dimensional data set consisting of the complex amplitudes at 17 frequencies. One can interpret such high-dimensional data sets with physical models or black-box models. Physical modelling uses analytic or numeric methods to understand the data in terms of underlying physical laws. Normal bimodal AFM, with its 4-dimensional data set (two frequencies) restricts physical interpretation to simple models^11^. The lack of a good method to calibrate the cantilever and measure the actual tip motion at the second eigenmode also limits the use of physical modelling in bimodal AFM^23^. In contrast, black-box modelling does not rely on a physical theory of the system being imaged, but rather attempts to draw conclusions from correlations in the raw data. Principle component analysis and neural networks have been applied to multidimensional AFM data^24^,^25^. We use Fisher's linear discriminant analysis (LDA)^15^ on two polymer components to demonstrate a quantitative improvement in image contrast by including response at harmonics and intermodulation products.

**Figure 3:**
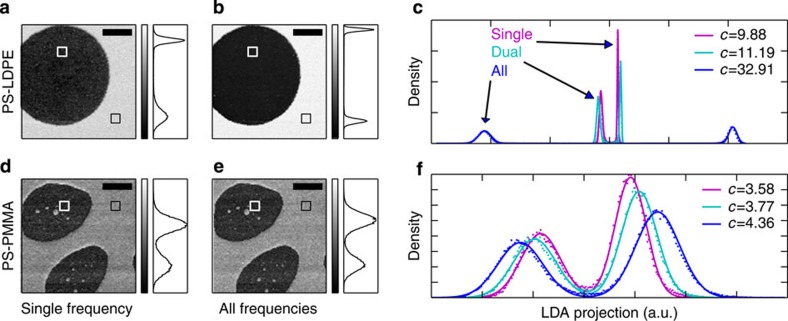
Linear discriminant analysis. PS–LDPE (**a**–**c**) and PS–PMMA (**d**–**f**). Images **a**,**b**,**d**,**e** are the result of projection with the LDA transform for two cases: single (**a**,**d**) and all (**b**,**e**). Squares show the areas used as training data and the scale bar is 500 nm. The grey scale is formed from the stretched histograms shown to the right of each image. Histograms are plotted on a common axis in **c** and **f** for all three cases: single (magenta) takes response at *f*_1_ only, dual (cyan) takes response at both *f*_1_ and *f*_2_, and all (blue) takes response at both drives and 15 additional nonlinear mixing frequencies. Fitting the histograms to a binormal distribution (lines) gives the contrast metric *c*, equation (3).

Each pixel is an independent measurement of 34 ‘features’ represented by a vector in the 34-dimensional space. We require that each pixel belong to one of two classes, for example, PS or LDPE. The LDA algorithm will find the linear combination of the measurement bases, which maximizes the separation between the two classes and minimizes the variance within the classes^26^. This optimal projection is calculated from manually selected training data having an assumed classification. A new image is formed by applying the optimal projection to all pixels (Fig. 3a,b,d,e). Three linear discriminant analyses were performed using image features defined in three different ways: one using response only at the two driven frequencies (denoted ‘dual’); one using all 17 frequencies (‘all’); and one using only the first drive frequency *f*_1_ (‘single’). This last analysis allows for comparison of bimodal AFM with standard single frequency AM-AFM. A separate scan performed with the second drive frequency turned off gave the same result as analysing only *f*_1_ with a bimodal drive.

Histograms for the projected images were fit to a binormal distribution and the contrast metric equation (3) was calculated (Fig. 3c,f). A small increase of contrast was found going from single frequency to bimodal AFM, 13% increase for the PS–LDPE and 5% increase for PS–PMMA. However, when including all frequencies in the analysis a much larger improvement in contrast was found: almost threefold for PS–LDPE and 15% increase for PS–PMMA. Similar improvements were found in multiple scans with several different cantilevers, including a much stiffer and higher Q-factor cantilever (BudgetSensor Tap300-Al, *k*=32 N m^−1^, *Q*_1_=548). These improvements were obtained from measuring response at additional non-driven frequencies, without changing experimental conditions such as the driving force or feedback set-point. Therefore, contrast was gained without increasing the applied force to the surface.

## Discussion

The LDA analysis shows that including high order intermodulation products enhances the ability to discriminate between the two components of the polymer blend. The image contrast is influenced by at least two factors: the physical process creating the response and noise when measuring the response. The cantilever is more sensitive to force on resonance, resulting in a higher signal-to-noise (SNR) ratio. However, the physical process that gives rise to contrast is the tip–surface force, which is a nonlinear perturbation of the cantilever dynamics. This nonlinear process can be much stronger off resonance, at harmonic and intermodulation frequencies.

Combining nonlinearity and resonance is most favourable. For instance, more mixing terms with larger SNR would occur if the second eigenmode resonance frequency was adjusted such that *D*=*f*_2_−6*f*_1_ was smaller. However, if the second resonance happened to be at exactly six times the first, it is not clear that choosing *f*_2_=6*f*_1_ would be advantageous. Driving at the 6th harmonic of *f*_1_ would not create mixing frequencies, only harmonics (integer multiples of *f*_1_). In this case it would be advantageous to drive the cantilever at *f*_1_ and *f*_2_=6*f*_1_±Δ*f*, where Δ*f* correspond to the measurement bandwidth for one pixel, such that many intermodulation frequencies occur close to resonance^6^.

Thus new cantilever designs could be used to enhance cantilever response^27^ and low noise detectors would help improve the SNR off resonance^28^,^29^,^30^. The enhanced contrast clearly seen at several mixing frequencies points towards further possible development of the methods used here. The use of black-box models such as LDA can help digest the high-dimensional data sets obtained with this and other emerging multifrequency AFM methods. For complex samples LDA extends to more than two classes and if the use of training data is not possible, there exists a large variety of unsupervised clustering algorithms, which could be applicable^31^. If calibration methods for higher modes are developed, physical models can be formulated, which take into account frequency mixing with multiple eigenmodes. Better quantitative analysis of the forces on the sample and therefore better discrimination of material composition and changes in topography will be possible. We recently described a method starting from an arbitrary physical model to approximate material properties (model parameters) using response at mixing frequencies^32^ and multiple eigenmodes^33^.

## Methods

### Sample preparation

The PS–LDPE sample was obtain from Bruker Corporation (HarmoniX test sample) and the PS–PMMA sample was made from polymers obtained from Sigma Aldrich. 17 mg PS (*M*_*w*_=280,000 kDa) and 52 mg PMMA (*M*_*w*_=120,000 kDa) were dissolved in 8 ml toluene and stirred for 4 h. A silicon substrate with native oxide was sonicated first in acetone, then isopropanol, followed by 4 min of O_2_ plasma. The polymer solution was spin cast on the substrate at 500 rpm for 5 s and 3000, rpm for 60 s, forming a ~20-nm thick layer.

### AFM imaging

AFM measurments were performed on a Nanowizard 3 BioScience AFM (JPK Instruments, Germany) with an additional multifrequency lockin amplifier^20^ (Intermodulation Products AB, Sweden). Thermal noise power spectra were used to determine the two first flexural eigenmode resonance frequencies and quality factors of a RFESPA AFM cantilever (Bruker): 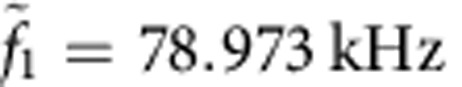, 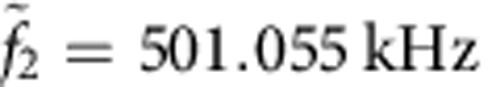 and *Q*_1_=183, *Q*_2_=485 respectively. The first mode was calibrated using the Sader–Higgins method^34^,^35^ to obtain a dynamic cantilever stiffness *k*_1_=2.5 N m^−1^ and the inverse photodetector responsivity *α*_1_^−1^=58 nm V^−1^. For the second mode ideal results from Euler-Bernoulli beam theory were used^17^ to estimate *k*_2_=39.32*k*_1_=99 N m^−1^ and *α*_2_^−1^=*α*_1_^−1^/3.573=17 nm V^−1^. Using this method the estimated quality factor of the second mode was a factor of 3 larger than the measured *Q*_2_. This discrepancy highlights difficulties in calibration of the second mode.

To avoid Fourier leakage, the drive frequencies *f*_1_=78.5 and *f*_2_=500.5 kHz were chosen as integer multiples of a common base frequency Δ*f*=500 Hz. Δ*f* constituted the measurement bandwidth or inverse of the measurement time window *T*=1/Δ*f*. The discrete Fourier transform or a lockin calculation (Fourier sum for one frequency) was performed over this time window. For *T*=2 ms per pixel and 256 pixels per line, the scan rate is ~1 line per second, typical for dynamic AFM imaging. Feedback was used to adjust the surface position so as to keep the amplitude at *f*_1_ constant

### Numerical methods

All histograms were normalized (total area equalled unity) and a sum of two normal distributions


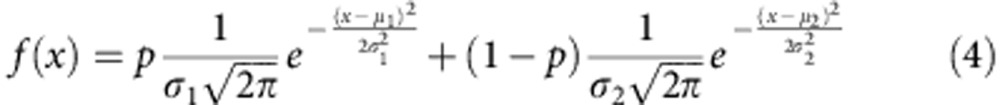


was fit to the histograms, where *μ*_1,2_ and 
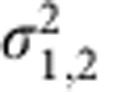
 are the mean and variances for the two distributions 1 and 2, *p* is the ratio of pixels in the first distribution and (1−*p*) the ratio of pixels in the second. The contrast metric was calculated from (3). Fisher's linear discriminant analysis was calculated using ref. 26 equation (4.30).

## Author contributions

D.F. conceived and performed the experiment, fabricated PS–PMMA sample and made the figures. D.F. and D.B.H. wrote the manuscript. D.F. and R.F. performed LDA analysis. All authors provided input to discussions and contributions to the manuscript. D.B.H. supervised the work.

## Additional information

**How to cite this article:** Forchheimer, D. *et al*. Improving image contrast and material discrimination with nonlinear response in bimodal atomic force microscopy. *Nat. Commun*. 6:6270 doi: 10.1038/ncomms7270 (2015).

## Supplementary Material

Supplementary Information Supplementary Figures 1-4 and Supplementary Tables 1-3

## Figures and Tables

**Table 1:** Observed contrast for driven, harmonics and mixing frequencies on PS–LDPE.

***f*** **(kHz)**	**Note**	**Type**	**Order**	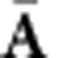 * **(mV)**	***c***_**real**_	***c***_**imag**_
78.5	*f*_1_	d	1	228.78	6.34	9.80
500.5	*f*_2_	d	1	34.81	7.22	6.47
157.0	2*f*_1_	h	2	0.37	8.45	5.14
235.5	3*f*_1_	h	3	0.15	2.75	4.24
314.0	4*f*_1_	h	4	0.09	2.54	4.79
392.5	5*f*_1_	h	5	0.25	8.54	2.85
471.0	6*f*_1_	h	6	0.51	16.71	3.38
549.5	7*f*_1_	h	7	0.20	8.26	7.05
422.0	*f*_2_−*f*_1_	m	2	0.22	5.31	0.60
579.0	*f*_2_+*f*_1_	m	2	0.20	0.96	1.85
29.5	*D*=*f*_2_−6*f*_1_	m	7	0.48	0.40	15.24
49.0	*f*_1_−*D*	m	8	0.53	2.33	7.69
108.0	*f*_1_+*D*	m	6	0.53	5.96	15.26
127.5	2*f*_1_−*D*	m	9	0.12	2.06	3.76
186.5	2*f*_1_+*D*	m	5	0.07	3.64	3.35
19.5	*f*_1_−2*D*	m	15	0.05	1.70	0.15
137.5	*f*_1_+2*D*	m	12	0.03	1.52	0.96

d, driven; h, harmonic; m, mixing tone; PS–LDPE, polystyrene and low-density polyethylene.

^*^Mean amplitude across the full image.
