# Supplementary material for: Improving image contrast and material discrimination with nonlinear response in bimodal atomic force microscopy
Source: Nat Commun. 2015 Feb 10;6:6270. doi: 10.1038/ncomms7270 (PMC4346977; doi:10.1038/ncomms7270)
Supplement: Supplementary Information — Supplementary Figures 1-4 and Supplementary Tables 1-3 [file ncomms7270-s1.pdf]

I. SUPPLEMENTARY FIGURES

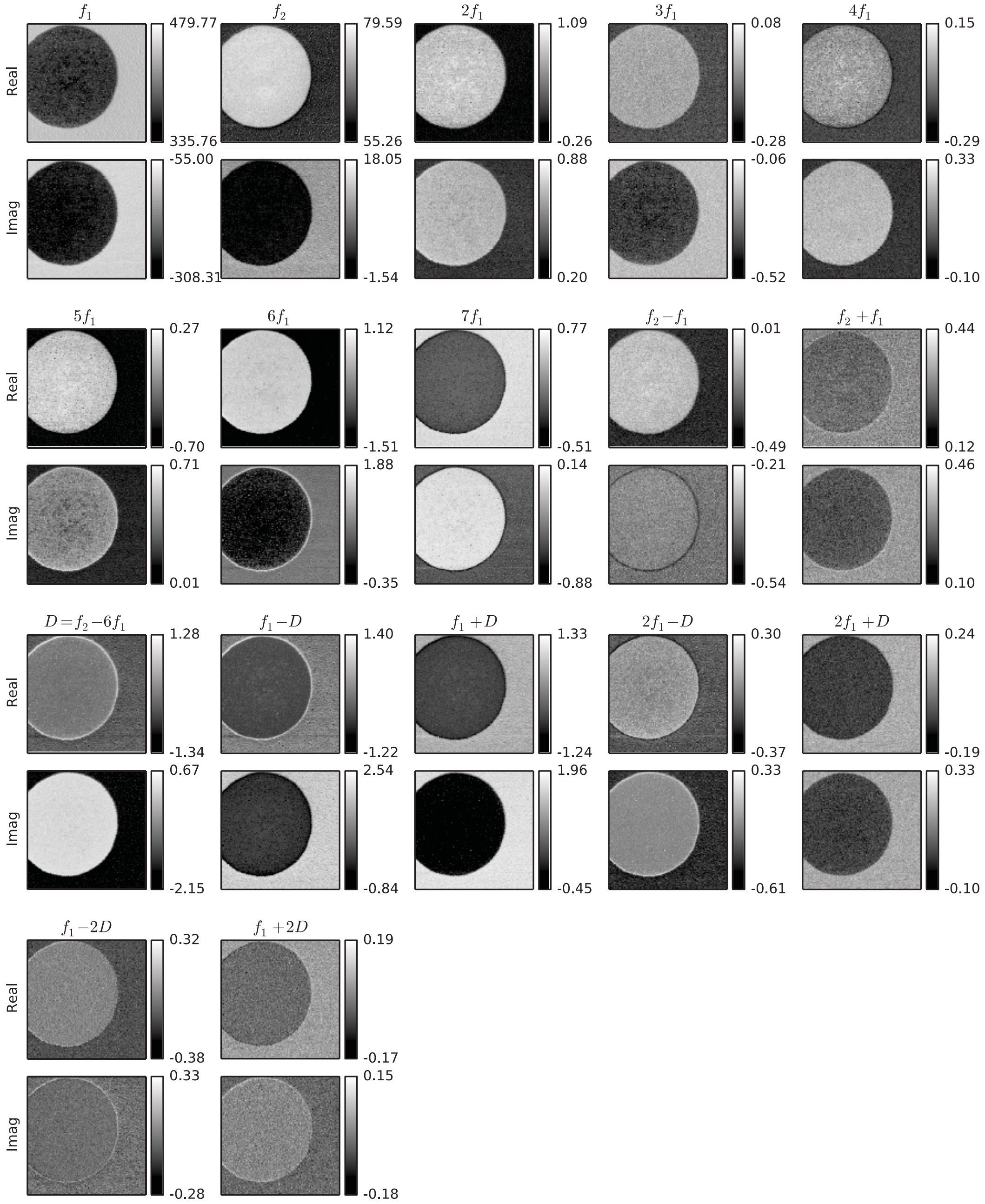

Supplementary Figure 1. Real and imaginary images for PS-LDPE

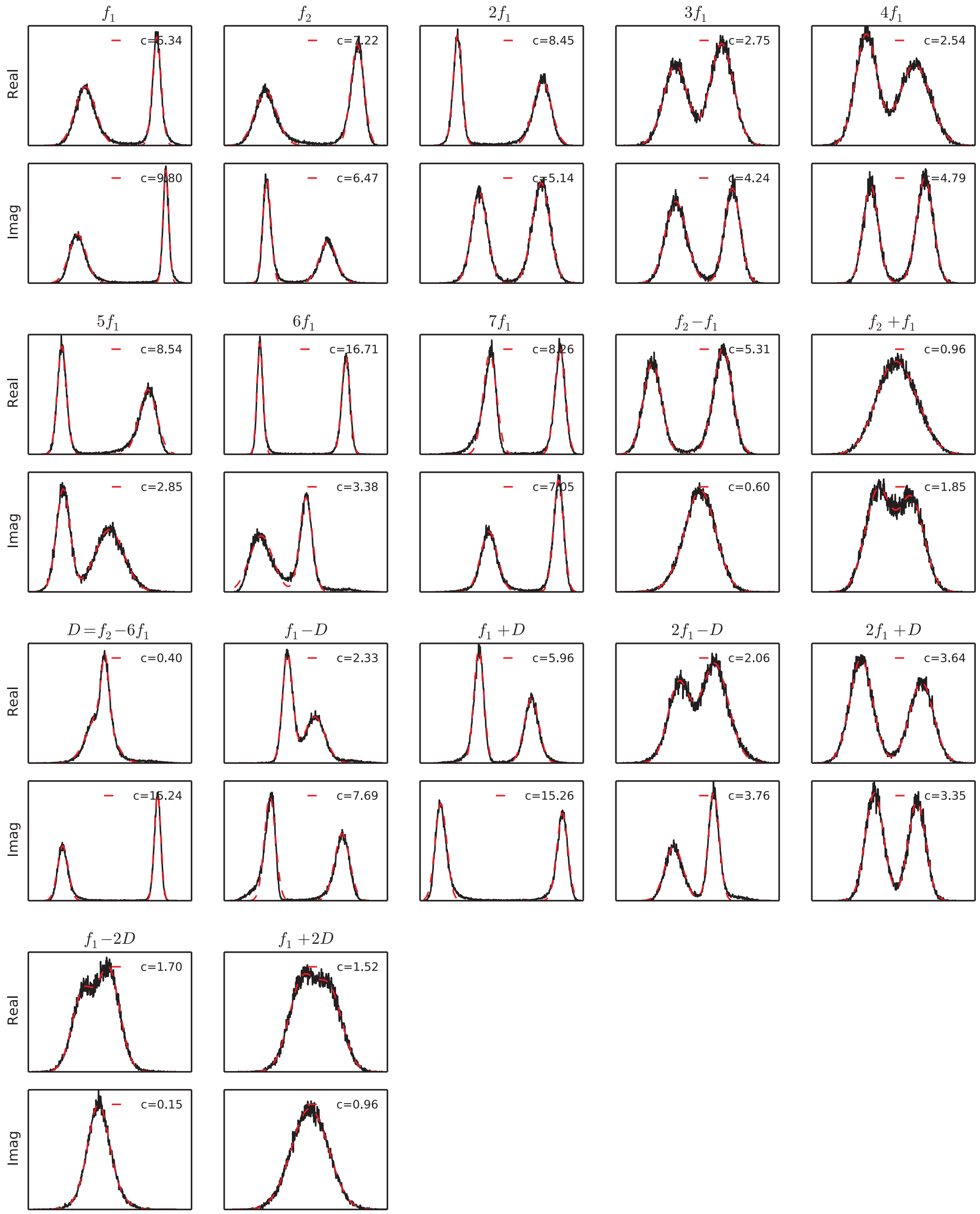

Supplementary Figure 2. Histograms and contrast fits for PS-LDPE

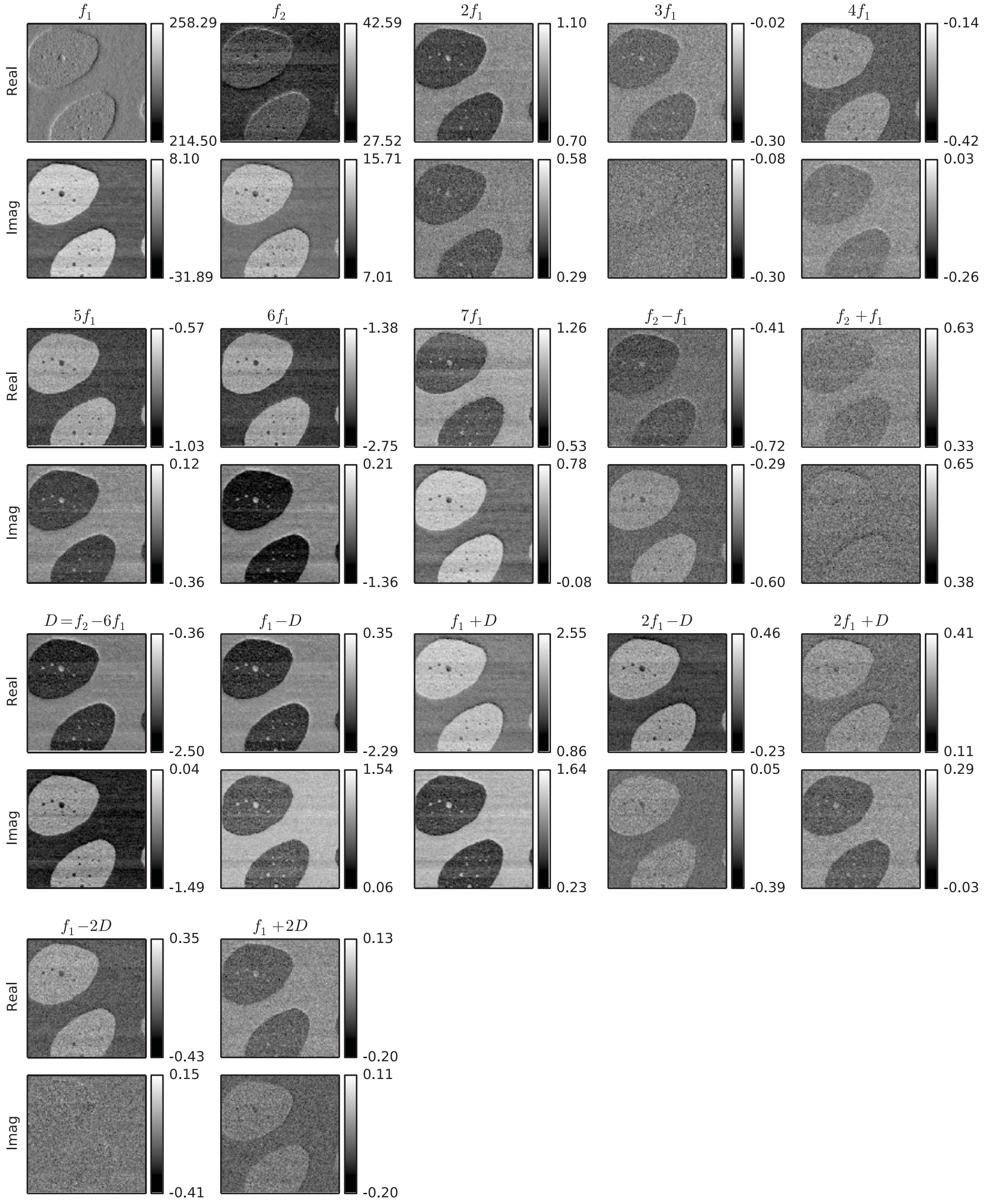

Supplementary Figure 3. Real and imaginary images for PS-PMMA

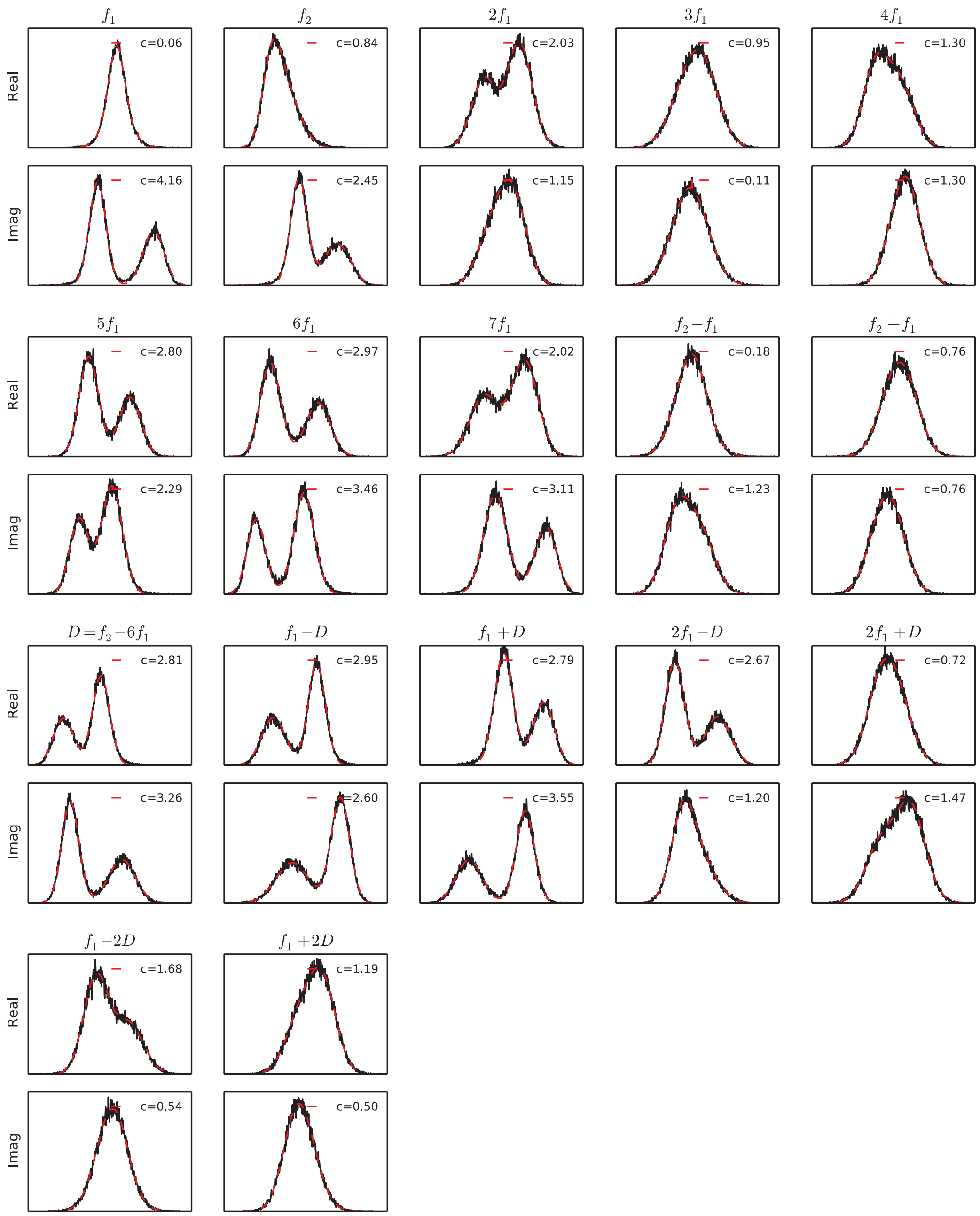

Supplementary Figure 4. Histograms and contrast fits for PS-PMMA

II. SUPPLEMENTARY TABLES

Supplementary Table I. Mean amplitudes A and phases ϕ calculated from the two 20x20 pixel training regions marked in manuscript Figure 3 for PS-LDPE

f (kHz)	Note	A_{LDPE} (mV)	A_{PS} (mV)	A_{LDPE}/A_{PS}	ϕ_{LDPE} (deg)	ϕ_{PS} (deg)	$\phi_{LDPE} - \phi_{PS}$ (deg)
78.5	f_1	228.83	228.49	1.00	-34	-12	-21
500.5	f_2	38.03	31.50	1.21	1	10	-9
157.0	$2f_1$	0.51	0.19	2.63	43	97	-54
235.5	$3f_1$	0.19	0.12	1.59	-96	-132	36
314.0	$4f_1$	0.11	0.08	1.42	98	69	29
392.5	$5f_1$	0.20	0.29	0.70	83	162	-79
471.0	$6f_1$	0.30	0.71	0.42	12	148	-136
549.5	$7f_1$	0.03	0.38	0.07	27	-40	67
422.0	$f_2 - f_1$	0.18	0.27	0.69	-108	-134	26
579.0	$f_2 + f_1$	0.17	0.22	0.76	43	46	-4
29.5	$D = f_2 - 6f_1$	0.13	0.90	0.15	101	-100	201
49.0	$f_1 - D$	0.18	0.96	0.19	74	86	-12
108.0	$f_1 + D$	0.22	0.89	0.25	-131	71	-202
127.5	$2f_1 - D$	0.05	0.20	0.25	-67	-107	39
186.5	$2f_1 + D$	0.04	0.11	0.35	81	61	20
19.5	$f_1 - 2D$	0.03	0.07	0.39	-10	10	-19
137.5	$f_1 + 2D$	0.02	0.04	0.54	9	-35	44

Supplementary Table II. Mean amplitudes A and phases ϕ calculated from the two 20x20 pixel training regions marked in manuscript Figure 3 for PS-PMMA. For this measurement an amplifier providing roughly a factor of 2 gain was removed, which explains the differences in voltage compared to Table I.

f (kHz)	Note	A_{PMMA} (mV)	A_{PS} (mV)	A_{PMMA}/A_{PS}	ϕ_{PMMA} (deg)	ϕ_{PS} (deg)	$\phi_{PMMA} - \phi_{PS}$ (deg)
78.5	f_1	118.58	118.67	1.00	0	-3	3
500.5	f_2	17.78	17.08	1.04	23	20	3
157.0	$2f_1$	0.46	0.50	0.91	26	26	0
235.5	$3f_1$	0.13	0.12	1.10	-135	-128	-7
314.0	$4f_1$	0.14	0.16	0.90	-154	-162	8
392.5	$5f_1$	0.37	0.43	0.88	-163	-172	9
471.0	$6f_1$	1.07	1.17	0.91	-149	-164	15
549.5	$7f_1$	0.50	0.52	0.96	38	22	16
422.0	$f_2 - f_1$	0.37	0.37	1.00	-146	-142	-4
579.0	$f_2 + f_1$	0.35	0.35	0.99	48	47	1
29.5	$D = f_2 - 6f_1$	1.04	0.89	1.17	-166	-146	-21
49.0	$f_1 - D$	0.89	0.69	1.30	161	131	30
108.0	$f_1 + D$	1.15	1.05	1.09	15	32	-16
127.5	$2f_1 - D$	0.13	0.09	1.42	-29	-87	58
186.5	$2f_1 + D$	0.15	0.15	1.03	17	30	-13
19.5	$f_1 - 2D$	0.09	0.10	0.89	-59	-121	62
137.5	$f_1 + 2D$	0.05	0.04	1.09	-90	-107	18

Supplementary Table III. Observered contrasts c_{real} and c_{imag} for PS-PMMA sample.

f (kHz)	Note	Type[a]	Order	Ā (mV)	c_{real}	c_{imag}
78.5	f_1	d	1	118.70	0.06	4.16
500.5	f_2	d	1	17.29	0.84	2.45
157.0	$2f_1$	h	2	0.48	2.03	1.15
235.5	$3f_1$	h	3	0.12	0.95	0.11
314.0	$4f_1$	h	4	0.15	1.30	1.30
392.5	$5f_1$	h	5	0.41	2.80	2.29
471.0	$6f_1$	h	6	1.14	2.97	3.46
549.5	$7f_1$	h	7	0.52	2.02	3.11
422.0	$f_2 - f_1$	m	2	0.37	0.18	1.23
579.0	$f_2 + f_1$	m	2	0.35	0.76	0.76
29.5	$D = f_2 - 6f_1$	m	7	0.95	2.81	3.26
49.0	$f_1 - D$	m	8	0.76	2.95	2.60
108.0	$f_1 + D$	m	6	1.09	2.79	3.55
127.5	$2f_1 - D$	m	9	0.11	2.67	1.20
186.5	$2f_1 + D$	m	5	0.15	0.72	1.47
19.5	$f_1 - 2D$	m	15	0.09	1.68	0.54
137.5	$f_1 + 2D$	m	12	0.04	1.19	0.50

[a] d - driven; h - harmonic; m - mixing tone
